# Supplementary material for: DAP12 deficiency alters microglia-oligodendrocyte communication and enhances resilience against tau toxicity
Source: Res Sq. 2023 Oct 26:rs.3.rs-3454358. Preprint. [Version 1] doi: 10.21203/rs.3.rs-3454358/v1 (PMC10635319; doi:10.21203/rs.3.rs-3454358/v1)
Supplement: Supplement 1 [file NIHPPRS3454358V1-supplement-1.pdf]

Supplementary Information:

DAP12 deficiency alters microglia-oligodendrocytes communication and enhances resilience against tau toxicity

Hao Chen[1], Li Fan[1], Qi Guo[2], Man Ying Wong[1], Fangmin Yu[1], Nessa Foxe[1], Winston Wang[3], Aviram Nessim[4], Gillian Carling[1,5], Bangyan Liu[1,5], Chloe Lopez-Lee[1,5], Yige Huang[1,11], Sadaf Amin[1], Sue-Ann Mok[6], Won-min Song[7], Bin Zhang[7], Flint Beal[8], Qin Ma[2], Hongjun Fu[9], Li Gan[1,3]*, Wenjie Luo[1]*

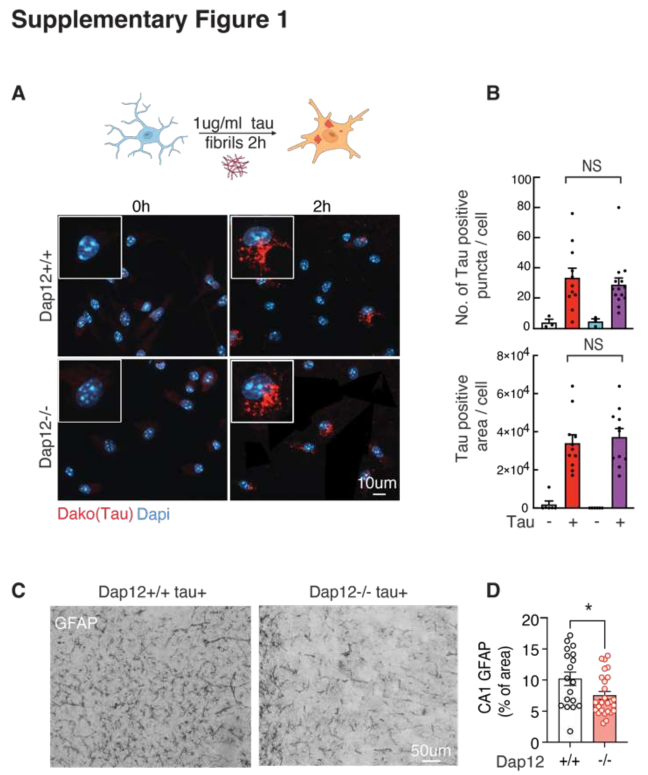

Supplementary Figure 1: Characterization of tauopathy mouse brain with Dap12 deficiency and the effect of Dap12 deletion on microglia tau phagocytosis, related to Figure1

A-B) Representative images (A) and quantification (B) of Tau positive puncta numbers and area in primary cultured microglia. Scale bar: 10 µm. Two-Way ANOVA with post hoc test, NS: Not significant. *n* = 11-14 from 3 independent experiments.

C-D) Representative immunohistochemical staining (C) and quantification (D) of GFAP+ area in the CA1 of *Dap12*$^{+/+}$ *Tau*+, and *Dap12*$^{-/-}$ *Tau*+ mice. Scale bar: 50 µm. Unpaired student's t-test: *p<0.05. n = 18 *Dap12*$^{+/+}$ *Tau*+, n = 26 *Dap12*$^{-/-}$ *Tau*+ mice.

Supplementary Figure 2

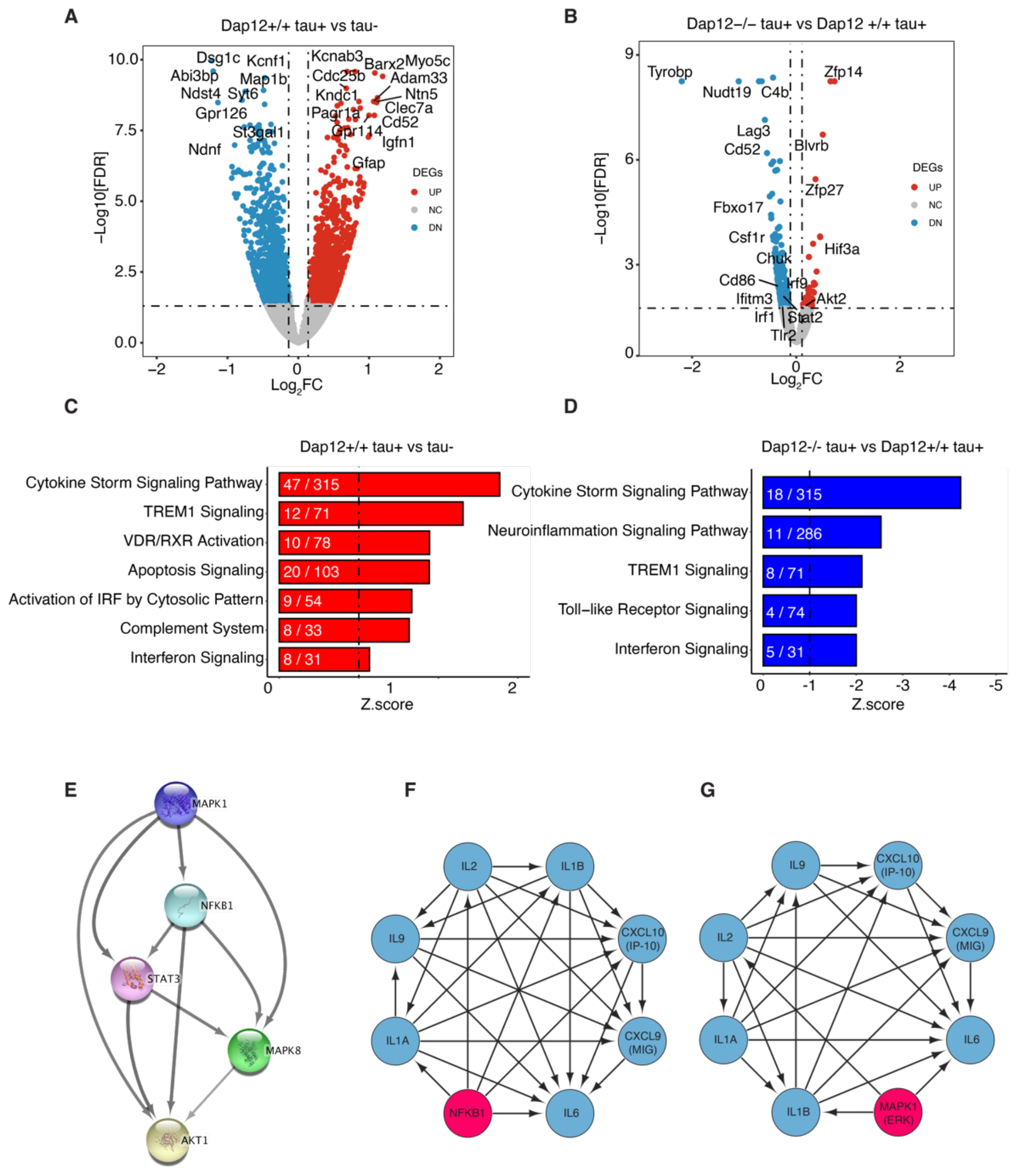

Supplementary Figure 2: Deletion of Dap12 suppressed inflammatory signaling in tauopathy mouse brain

A-B) Volcano plot of DEGs (adjust p-value < 0.05, Log_2FC > 0.1 or < -0.1) comparing *Dap12*$^{+/+}$ *Tau*+ versus *Tau*- mice (A) and *Dap12*$^{-/-}$ *Tau*+ mice versus *Dap12*$^{+/+}$ *Tau*+ mice.

C-D) Selected top IPA canonical pathways identified from the DEGs of *Dap12*$^{+/+}$ *Tau*+ vs *Tau*- mice (C) or *Dap12*$^{-/-}$ *Tau*+ vs *Dap12*$^{+/+}$ *Tau*+ mice (D). IPA canonical pathways contain z score and -log10(p-value). No log2FC or adjust p-value.

E) String gene network analysis showing relationships between immune regulators identified in Figure 2K.

F-G) String gene network analysis showing cytokines regulated by NF-κB (F) or ERK (G).

Supplementary Figure 3

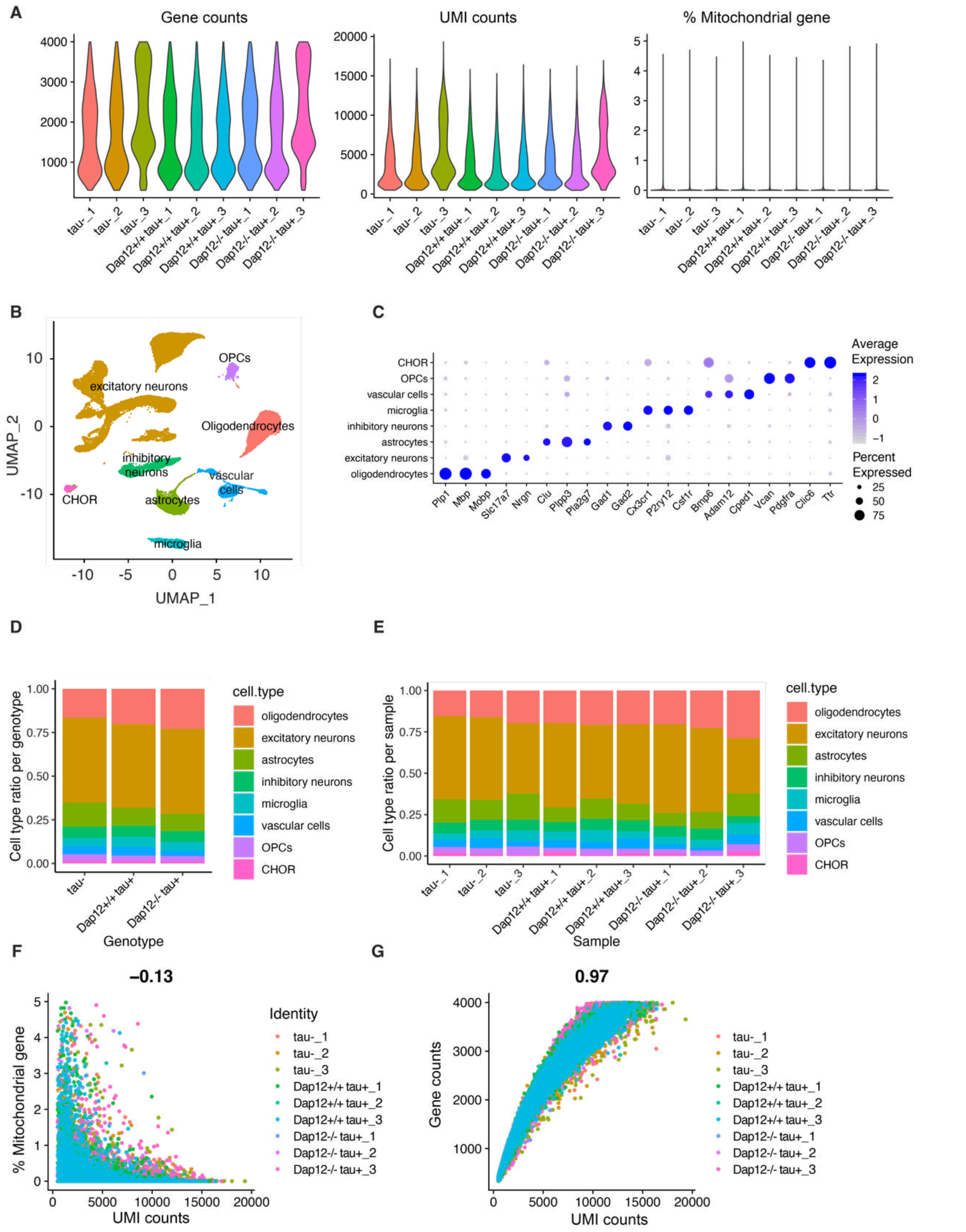

Supplementary Figure 3: Quality control assessment of Single-Nuclei RNA-seq (related to Fig. 3-6)

A) Quality control plots showing equivalent amounts of total number of genes, total number of molecules and percent mitochondrial RNA in nuclei used for downstream analyses.

B) UMAP dimensional plot showing nuclei colored according to transcriptionally distinct cell clusters identified using Seurat package.

C) Summary of genes used for cluster classification into different cell types.

D) Proportion of each cell type detected across the different genotypes.

E) Proportion of each cell type detected across the different samples.

F-G) Correlation between UMI counts and percentage of mitochondrial genes per nuclei (F) and total genes detected (G) for all samples.

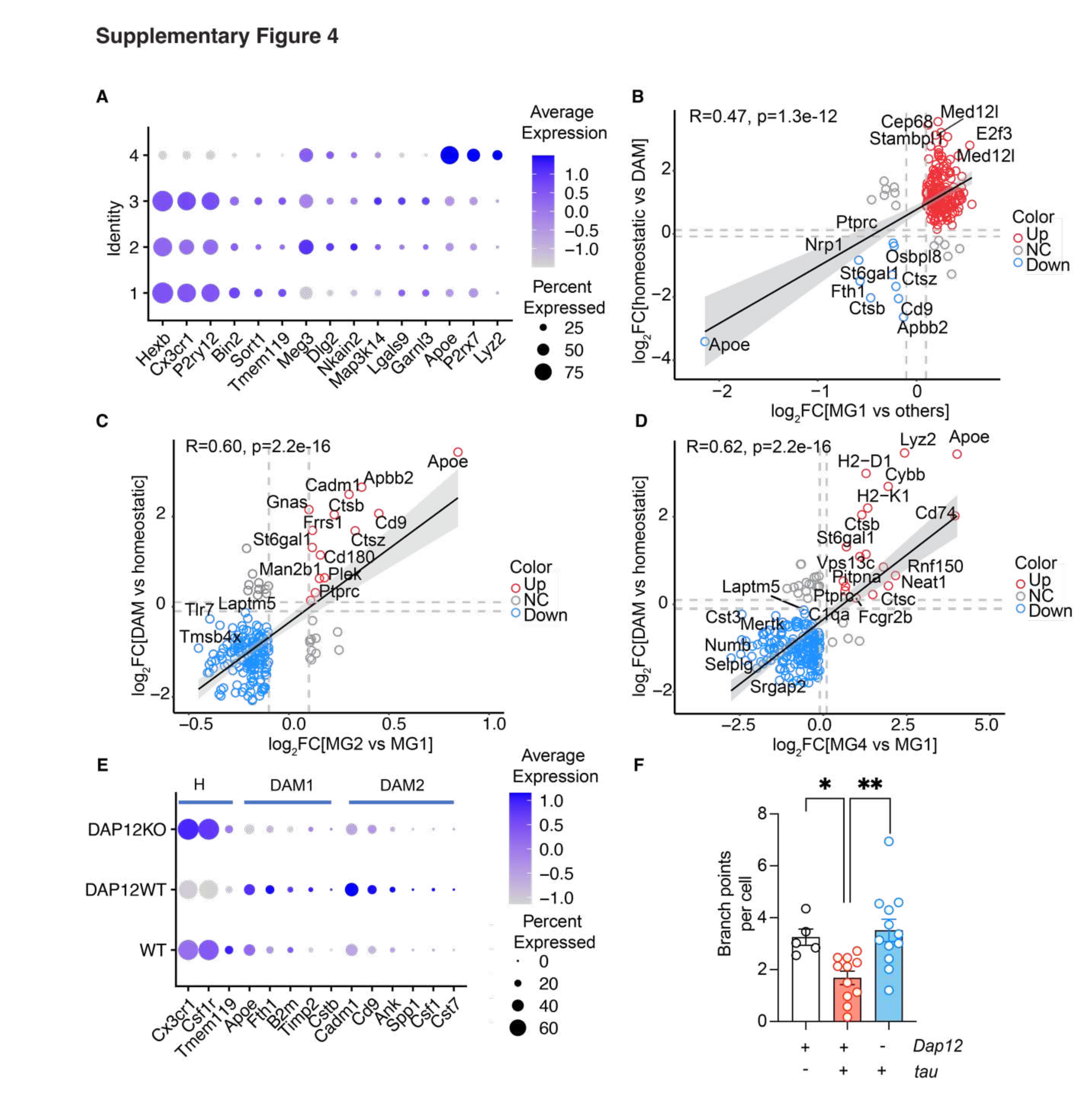

Supplementary Figure 4: Characterization of microglia clusters (Related to Figure 3).

A) Dot plot of marker genes crossing different MG clusters.

B) Correlation scatterplot of marker genes comparing MG1 cluster versus other clusters.

C) Correlation scatterplot of marker genes comparing MG2 versus MG1 cluster.

D) Correlation scatterplot of marker genes for MG4 versus MG1 cluster.

E) Dot plot of homeostatic and DAM marker genes in crossing different genotypes.

F) Quantification of microglial branch points crossing three genotypes. n = 5 *tau*-, n = 11 *Dap12+/+ tau+*, n = 12 *Dap12-/- tau+* mice.

Supplementary Figure 5

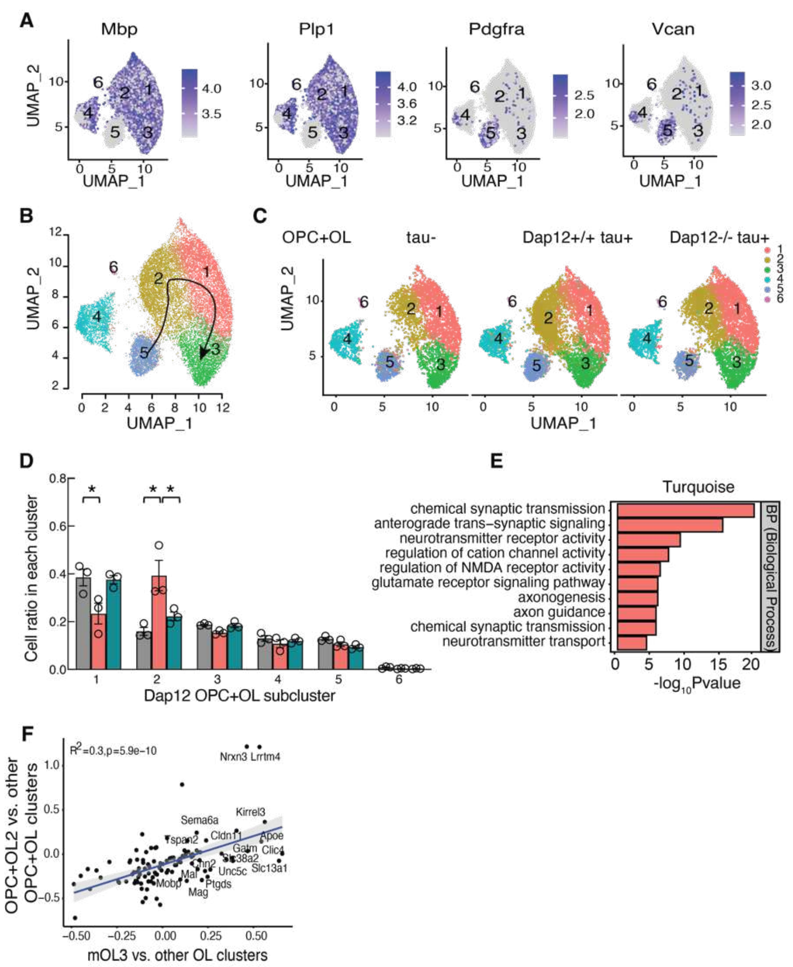

Supplementary Figure 5. Dap12 is essential for tau-induced transcriptomic changes in oligodendrocyte lineage cells in vivo

A) Feature plots of marker genes for OPC (Pdgfra and Vcan) and OL (Mbp and Plp1) subclusters.

B) Slingshot showing transition between OL lineage cell subclusters.

C) UMAP of integrated OL lineage clusters with integrated OPC and OL.

D) Cell ratios of OPC+OL subclusters (1,2,3,4,5 and 6) crossing three genotypes. One-Way ANOVA followed by turkey test, ***p<0.001, **p<0.01, *p<0.05. *n* = 3 per genotype.

E) Bar chart of the enriched pathways for the signature gene sets of the Turquoise module identified by Gene Ontology (GO) pathways.

F) Correlation scatterplot of DEGs between integrated OPC+OL2 and mouse oligodendrocyte cluster 3 (mOL3)

Supplementary Figure 6

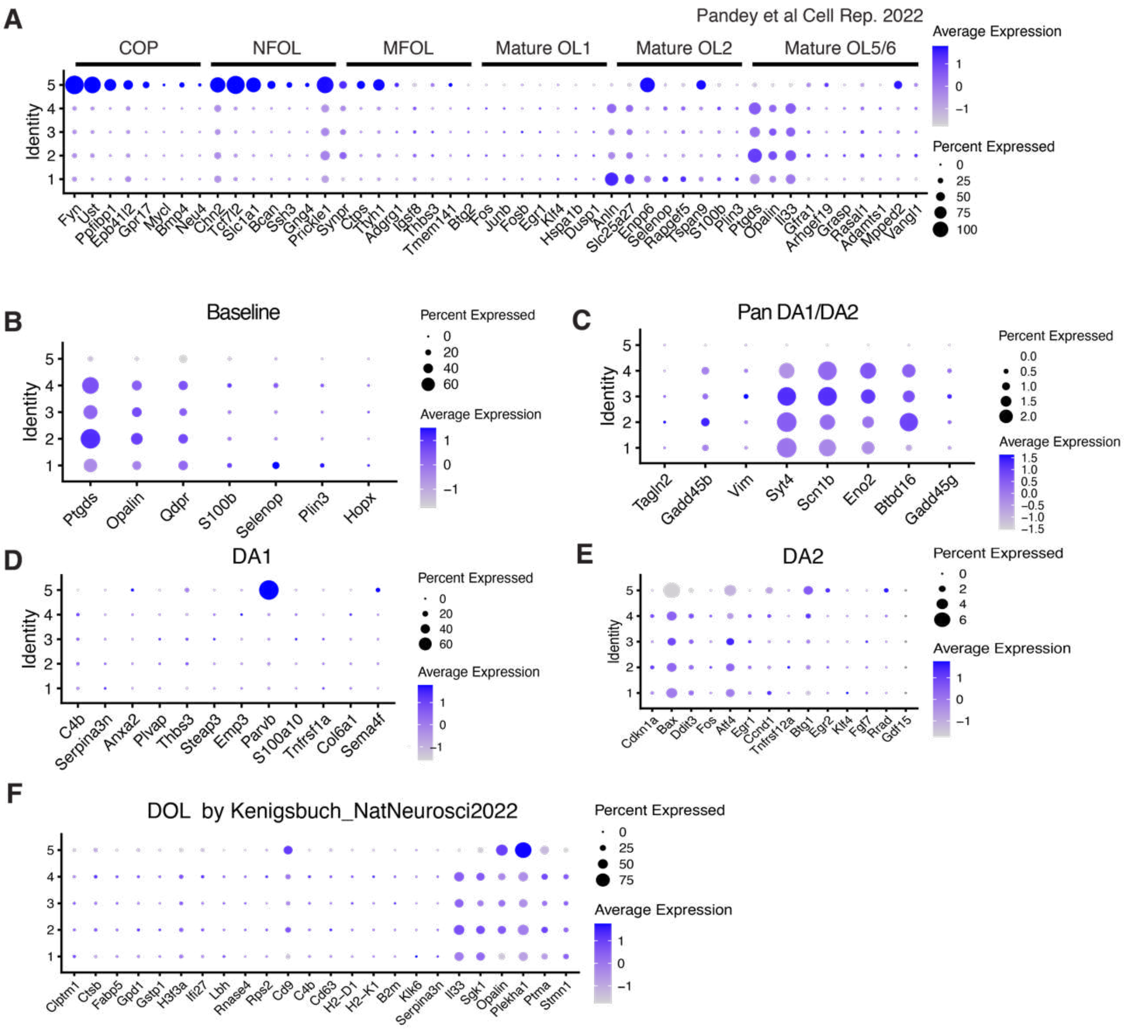

A) Dot plot of marker genes of previous identified oligodendrocyte clusters.

B-E) Dot plot of marker genes for baseline (B), pan disease associated oligodendrocyte marker genes (C), disease associated stage 1 oligodendrocyte (D), and disease associated stage 2 oligodendrocyte(E).

F) Dot plot of marker genes of disease associated oligodendrocyte identified in previous report.

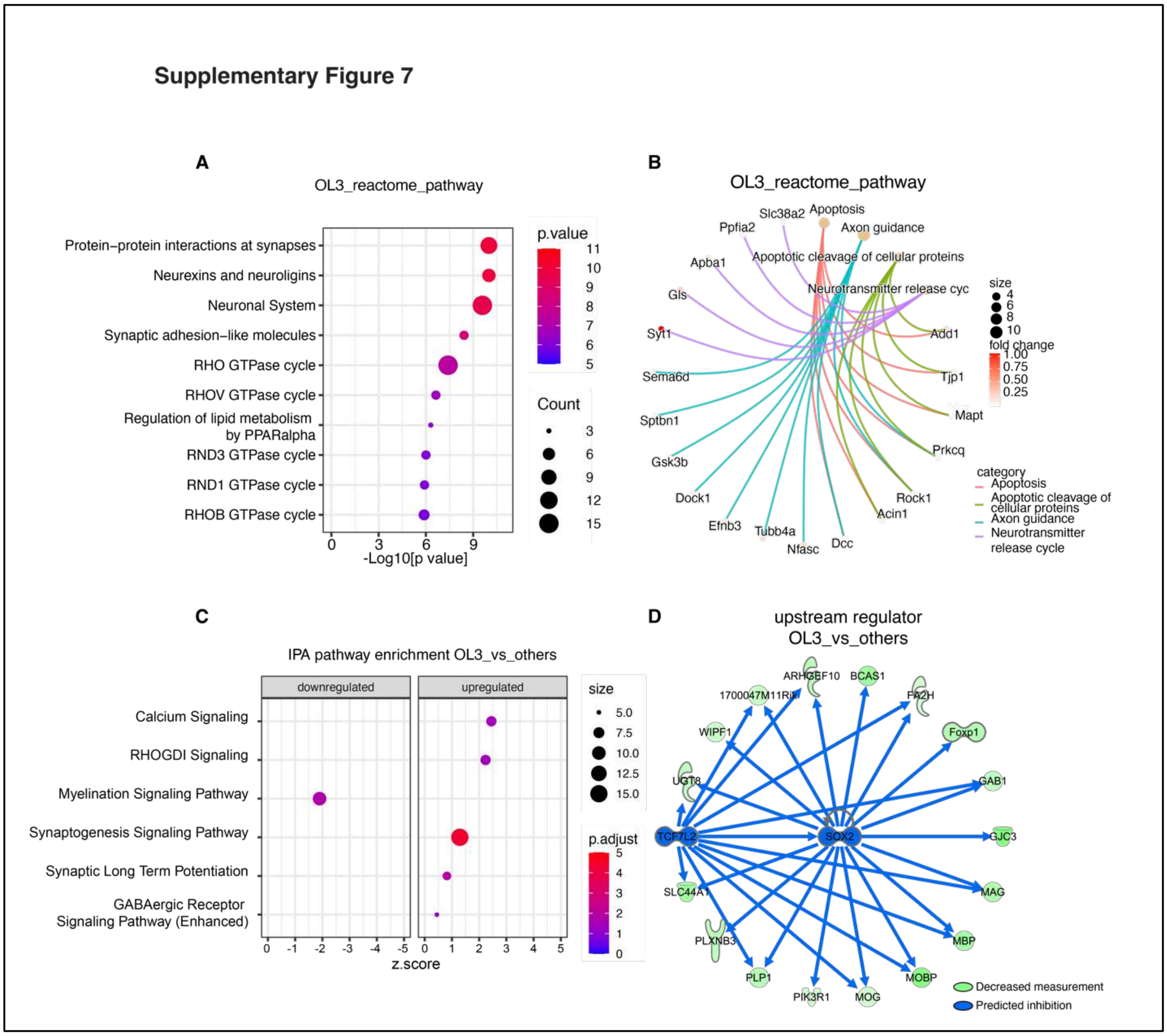

Supplementary Figure 7: Characterization of oligodendrocyte clusters

A-B) Reactome pathways enriched in DEGs identified in OL3 (A) and selected top reactome pathways with associated genes (B).

C) Selected IPA canonical pathways identified for DEGs of OL3.

D) Tcf7l2 and Sox2 predicted by IPA as upstream regulators of a subset of DEGs downregulated in OL3.
